# Recent Research on the Health Benefits of Blueberries and Their Anthocyanins

**DOI:** 10.1093/advances/nmz065

**Published:** 2019-07-22

**Authors:** Wilhelmina Kalt, Aedin Cassidy, Luke R Howard, Robert Krikorian, April J Stull, Francois Tremblay, Raul Zamora-Ros

**Affiliations:** 1 Agriculture and Agri-Food Canada, Kentville Research and Development Centre, Kentville, Nova Scotia, Canada (retired); 2 Department of Nutrition, Norwich Medical School, University of East Anglia, Norwich, United Kingdom; 3 Department of Food Science, University of Arkansas, Fayetteville, AR, USA; 4 Department of Psychiatry and Behavioral Neuroscience, University of Cincinnati Academic Health Center, Cincinnati, OH, USA; 5 Department of Human Ecology, University of Maryland Eastern Shore, Princess Anne, MD, USA; 6 Department of Ophthalmology and Visual Sciences and Department of Physiology and Biophysics, Dalhousie University, Halifax, Nova Scotia, Canada; 7 Unit of Nutrition and Cancer, Cancer Epidemiology Research Programme, Catalan Institute of Oncology, Bellvitge Biomedical Research Institute (IDIBELL), Barcelona, Spain

**Keywords:** anthocyanin, berries, cardiovascular, cognition, diabetes, obesity, processing, vision

## Abstract

Awareness of the human health benefits of blueberries is underpinned by a growing body of positive scientific evidence from human observational and clinical research, plus mechanistic research using animal and in vitro models. Blueberries contain a large number of phytochemicals, including abundant anthocyanin pigments. Of their various phytochemicals, anthocyanins probably make the greatest impact on blueberry health functionality. Epidemiological studies associate regular, moderate intake of blueberries and/or anthocyanins with reduced risk of cardiovascular disease, death, and type 2 diabetes, and with improved weight maintenance and neuroprotection. These findings are supported by biomarker-based evidence from human clinical studies. Among the more important healthful aspects of blueberries are their anti-inflammatory and antioxidant actions and their beneficial effects on vascular and glucoregulatory function. Blueberry phytochemicals may affect gastrointestinal microflora and contribute to host health. These aspects have implications in degenerative diseases and conditions as well as the aging process. More evidence, and particularly human clinical evidence, is needed to better understand the potential for anthocyanin-rich blueberries to benefit public health. However, it is widely agreed that the regular consumption of tasty, ripe blueberries can be unconditionally recommended.

## Introduction

Blueberries were first popularized as a “super fruit” due mainly to the high in vitro antioxidant capacity of their abundant polyphenolic compounds. However, direct antioxidant action of polyphenolic compounds in situ appears unlikely due to their poor bioavailability (1). Nonetheless, research regarding foods for health performed during the past 2 decades has revealed a multitude of ways in which blueberries are bioactive and beneficial to health.

An increasing body of evidence suggests that blueberries and anthocyanins reduce biomarkers and risk of diseases that constitute major socioeconomic burdens, including cardiovascular disease (CVD), type 2 diabetes mellitus (T2DM), and neurological decline. In these observational analyses, anthocyanins often provide benefits over and above other plant food phytochemicals, including other flavonoids (2–6). The intake of even moderate amounts of blueberries (approximately one-third cup) and anthocyanins (<50 mg) daily is associated with disease risk reduction (2–4, 6–9).

In this narrative, research on the role of blueberries in cardiometabolic health, neuroprotection, vision, and food processing is reviewed. Observational evidence is presented along with results from human clinical studies, and from animal and in vitro research. Over half of the nearly 200 papers cited in this review were published in the last decade. Blueberry research is the primary focus of this review; however, anthocyanin literature is also discussed where relevant. Interest continues to grow in the potential human health benefits of blueberries.

## Current Status of Knowledge

### Blueberry species and composition

Blueberry species of commercial importance include highbush blueberry (*Vaccinium corymbosum* L.), rabbiteye blueberry (*V. virgatum* Aiton), lowbush blueberry (*V. angustifolium* Aiton), and European bilberry (*V. myrtillus* L.). Blueberries are one of the richest sources of anthocyanins among common fruits (10–12) (**Table 1**). Anthocyanins are the pigments that confer the red, blue, and purple coloration to ripe berries. During berry ripening, anthocyanin content rises dramatically to provide a visual cue to distinguish between early to fully ripe fruit (13).

**TABLE 1:** Total anthocyanin concentration of popular fruit consumed in the United States

Fruit	Description	Number of samples	Total anthocyanins (mg/100 g fresh)
Apple^1^	Red peel	6	12
Apple^1^	Yellow peel	2	0
Banana^2^		—	0
Blackberry^2^		4	245
Blueberry^2^	Highbush	7	387
Blueberry^2^	Lowbush	1	487
Cantaloupe^2^		—	0
Cherry (sweet)^2^		4	122
Grape^2^	Red peel and flesh	5	27
Grape^2^	Purple peel and flesh	1	120
Kiwifruit^2^		—	0
Nectarine^3^	Yellow peel	5	15
Orange^2^	Orange flesh	—	0
Plum^3^	Yellow peel	1	0
Plum^3^	Red peel	2	20
Plum^3^	Black peel	2	116
Raspberry (red)^2^		5	92
Strawberry^2^		8	21
Watermelon^2^		—	0

1 Reference 11.

2 Reference 10.

3 Reference 12.

Among a selection of 80 highbush and 135 lowbush blueberry phenotypes, 90% of the phenotypes spanned a 1.6-fold range in anthocyanin concentration (14). Among the total 215 blueberry phenotypes, the range between the 10th and 90th percentiles of cyanidin-3-glucoside equivalents/g fresh weight was 0.925 to 2.1 mg (14).

### Blueberry polyphenolic compounds

Anthocyanin flavonoids account for up to 60% of the total polyphenolics in ripe blueberries (13). Therefore, anthocyanins probably make the greatest contribution to blueberry health benefits. Blueberry polyphenolic compounds include both flavonoid and nonflavonoid types. Other classes of flavonoids found in blueberries include proanthocyanidins (15, 16) and flavonols (17, 18). Abundant nonflavonoid polyphenolic compounds in blueberries are the hydroxycinnamic acid esters (especially chlorogenic acid) (16, 17, 19, 20).

### Anthocyanin bioavailability

Associating the in vivo metabolites of anthocyanins with health outcomes has been difficult. After ingestion, anthocyanins are converted to a large number of products via chemical events and human and microbial metabolism. Clearance time for anthocyanin metabolites varies widely (21, 22). To illustrate, within 6 h after humans ingested ^13^C-labeled anthocyanin, substantial ^13^C-labeled CO_2_ was detected in exhaled breath, which demonstrated rapid and complete anthocyanin catabolism. However, >50% of the ^13^C still remained in the body after 48 h (21). Anthocyanins and their phase 2 metabolites persist in urine long after anthocyanin intake (23), probably due to their transport in bile (24, 25). Also, anthocyanins and their metabolites become localized in body tissues (24, 26–29). Due to the catabolic action of gastrointestinal microflora on anthocyanins and other food polyphenolics, phenolic acid products are very abundant in the large intestine (30).

## Cardiovascular Health

### Population studies in cardiovascular health, berries, and anthocyanins

The association between a higher anthocyanin intake and reduction in all-cause mortality risk in a meta-analysis of 6 studies was principally due to a decreased cardiovascular mortality risk (31). Similar findings were reported in a meta-analysis of total CVD (RR: 0.89; 95% CI: 0.83–0.96) (32). In 3 cohort studies, a higher anthocyanin intake was associated with an ~25% reduced risk of coronary artery disease, including fatal and nonfatal myocardial infarction (33, 34). Higher intakes of blueberries, strawberries, and total anthocyanins were all associated with a 32% lower rate of myocardial infarction, and this association was independent of established risk factors (2). However, in 2 prospective cohort studies no association was found between anthocyanin intake and stroke risk (34, 35).

Higher anthocyanin intake was associated with an ~8–10% reduction in hypertension risk in 5 cohort studies (3, 36, 37). A higher anthocyanin intake was associated with a 10% lower risk of incident hypertension in a cohort of over 87,000 participants examined over a period of 14 y (3). The greatest risk reduction was observed in women aged ≤60 y (3). One biomarker, vascular stiffness, was measured in a cross-sectional study of 1898 carefully phenotyped twins. In this study, a clinically relevant improvement in vascular modulation, measured using pulse wave velocity, was associated with greater anthocyanin intake (6).

### Population studies in CVD, obesity, berries, and weight maintenance

Obesity and overweight are major contributors to CVD risk (38). Even minor weight gain can increase the risk of hypertension (39) and CVD (40, 41). Reducing BMI by 1–3 kg/m^2^ was associated with a 2–13% lower risk of CVD events (41) and mortality (42). In a comparison of intakes of 16 common fruits, the highest blueberry intake was associated with the least weight gain (−0.64 kg over 4 y) in a prospective study of over 133,000 men and women followed for ≤24 y (43). Among 6 classes of flavonoids, a higher anthocyanin intake had the strongest association with less weight gain (−0.1 kg per 10 mg anthocyanins) in a study of 124,000 individuals (44).

Greater anthocyanin intake was associated with 3–9% lower fat mass and less central adiposity in healthy female twins (*n *= 2734) (45) based on body composition assessment using DXA (46). In this study, the twin with the higher blueberry intake had a lower fat mass ratio than the co-twin (45). Results of the twin studies are most interesting because they are independent of genetic and common environmental factors.

### Clinical studies in cardiovascular health

In clinical research on blueberries, subjects most often have some CVD risk (e.g., metabolic syndrome markers, T2DM). In a placebo-controlled study of 58 diabetic patients, blueberry intake led to a decline in LDL cholesterol, triglycerides, and adiponectin and an increase in HDL cholesterol (47). Intake of purified anthocyanin for 12 wk by 150 hyper-cholesterolemic subjects was associated with an increase in HDL cholesterol and a decrease in LDL cholesterol as well as improved endothelial function (brachial flow-mediated dilation) (48). Then, after 24 wk of anthocyanin intake by the same 150 hypercholesterolemic patients, a reduction was documented in inflammatory markers, including serum high-sensitivity C-reactive protein, soluble vascular adhesion molecule-1, and plasma IL-1β (49).

Arterial stiffness was reduced and both systolic and diastolic blood pressure were decreased by 5–6% after 8 wk of blueberry intake in women with pre- and stage 1 hypertension (50, 51). Similar benefits were observed in middle-aged unmedicated men with CVD risk factors (51). In subjects with metabolic syndrome, vascular endothelial function was improved although blood pressure was unaffected by blueberry intake for 6 wk (52). In a blueberry study examining participants with metabolic syndrome (*n *= 115), after 6 mo of taking either 0, 75, or 150 g, biomarkers of cardiometabolic function were unchanged in the group taking 75 g blueberries daily. However, the group taking 150 g blueberries daily had sustained improvements in vascular function and lipid status. Insulin resistence was not affected by either dose of blueberries (53). Some clinical studies have reported little to no effect of blueberry intake on blood pressure (54, 55). In contrast to these long-term studies, in a 6-h acute study design, blueberry intake was associated with short-term improvements in vascular function measured by flow-mediated dilation in 21 healthy men (56).

### Mechanisms of cardiovascular benefit

Blueberries and anthocyanins benefit cardiovascular health via antioxidant and anti-inflammatory effects (49, 57) positive effects on plasma lipid levels, and modulation of glucose metabolism and endothelial function (see reviews, 58, 59). Blueberries protect vasculature in various ways that can be detected by vascular responsiveness, blood pressure, and arterial stiffness (18, 50–52, 60). These benefits may involve NO metabolism (53, 61) and effects on endothelium composition (62) and plasma lipids (47, 48, 63). Most often, cardiovascular research models employ a relevant stress treatment (e.g., diet or disease) or examine a population with existing risk condition(s).

Nonflavonoid catabolites of berry anthocyanins predominate in the large intestine (1) and could interact with the microbiota to elicit anti-inflammatory or other responses that contribute to cardioprotective benefits (64). Blueberry supplementation modified the colonic microflora of rats (65, 66). By use of gene sequencing, 3 new phyla and 22 new genera of micro-organisms were found to be specifically associated with blueberry feeding (66). These gene changes accounted for ~9% of the entire genome and were associated with species in the intestinal mucin layer, as well as better protection from bacterial invasion and greater capacity for xenobiotic metabolism (66). In a study with high-fat–fed rats, blueberry intake moderated the negative effects of the high-fat diet on inflammation and insulin signaling and also led to modification of the gut microbiota (67).

## Prediabetes and T2DM

### Population studies in T2DM, blueberries, and anthocyanins

Prediabetes and T2DM affect ~100 million adults in the United States (68). Both prediabetes and T2DM are characterized by poor response to insulin stimulation (i.e., insulin resistance) leading to inefficient glucose uptake and metabolism in insulin-sensitive tissues (69). Of all the fruits analyzed in 3 prospective studies, blueberries provided the strongest association, with T2DM risk reduction of 26% (RR: 0.74; 95% CI: 0.66–0.83) (70). In the same cohorts, when the intake of habitually consumed flavonoids (flavonols, flavones, flavanones, flavan-3-ols, and anthocyanins) was examined, intake of anthocyanins and particularly blueberries provided a similar degree of risk reduction of 23% with consumption of  ≥2 servings weekly or ≤1 serving monthly. There was no association found between the intake of total flavonoid or other flavonoid groups and reduced T2DM risk (4).

A meta-analysis of data from 3 US cohorts associated T2DM risk reduction with higher intake of anthocyanins (RR: 0.85, 95% CI: 0.80–0.91) and berry fruits (RR: 0.82, 95% CI: 0.76–0.89) (71). A similar association between T2DM risk reduction with greater anthocyanin intake was determined in a Polish cohort (RR: 0.68, 95% CI: 0.48–0.98) (72). In a cross-sectional study in women, higher habitual intake of anthocyanins and flavones was associated with improvements in insulin resistance, whereas only anthocyanin was associated with a decrease in inflammation and high-sensitivity C-reactive protein (8). Obesity is positively associated with T2DM risk (73). Greater blueberry and anthocyanin intake is associated with less weight gain during aging (43–45) and therefore would support reduced T2DM risk. Notably, not all observational studies identified an association of anthocyanin or berry intake with reduced T2DM risk (74, 75).

### Clinical studies in T2DM

In a placebo-controlled study of obese, insulin-resistant adults, insulin sensitivity was greater after 6 wk of blueberry intake (76). Insulin sensitivity was assessed using a hyperinsulinemic-euglycemic clamp, which directly measures whole-body glucose disposal (77).

Anthocyanin extract from bilberry and black currant (80 mg daily) improved insulin sensitivity (HOMA-IR), plasma lipid profiles, and reduced plasma markers of oxidative stress among 58 T2DM patients compared to placebo (47). When glucose-modulation effects were examined in a T2DM population after a single oral dose of either placebo or 0.47 g standardized bilberry extract containing 36% (w/w) anthocyanins, bilberry intake lowered plasma glucose and insulin AUC in the oral glucose tolerance test (78). In a 12-wk trial of 54 overweight young adults, replacing 50 g carbohydrate with 50 g blueberries daily produced favorable reductions in body weight (BW), insulin, cholesterol, and other metabolic factors (63).

### Animal and mechanistic studies in T2DM

Rodents with a phenotype and metabolic features of prediabetes and T2DM, plus diet-induced obesity, are often used to investigate mechanisms of action. C57BL/6 mice fed a high-fat (60%) diet for 8 wk had better insulin sensitivity when blueberries were added to the high-fat diet (79). Also, the glucose AUC of the mice fed a high-fat diet plus blueberries was similar to that of mice fed the low-fat diet (79).

In a study where Zucker fatty rats were fed a high-fat (45%) or low-fat (10%) diet, after 12 wk rats receiving a high-fat diet plus 2% blueberries and those fed a low-fat diet had better metabolic markers than mice fed a high-fat diet without blueberries. At that time rats fed a high-fat diet plus blueberries had better measures of fasting insulin levels, insulin resistance (HOMA-IR), and glucose AUC than high-fat–fed controls (80). Blueberry intake reduced markers of metabolic syndrome and adiposity in high-fat–fed, obesity-prone rats (80).

Insulin resistance (HOMA-IR) and glucose tolerance in obese mice were improved after 12–15 wk of diet supplementation with blueberries (81–83). Obese hyperglycemic mice that consumed blueberry powder that was sorbed and concentrated on defatted soybean flour had improved oral glucose tolerance and fasting glucose concentration, compared to controls (83).

Several but not all biomarkers of glucose metabolism were normalized by blueberry intake in obese Zucker rats (84). In other obese rodent studies, blueberry intake improved glucose tolerance (85) or not (86), and in some studies insulin responses were not improved (65, 84, 85, 87). However, in high-fat–fed mice, inflammatory markers and hypertension that are associated with obesity were mitigated (87).

Berry intake supports the growth of favorable mucin-producing bacteria that can protect of the lining of the gastrointestinal tract, which may mitigate lower intestinal and systemic inflammation and improve metabolic outcomes (88, 89).

## Neuroprotection, Cognition, and Blueberries

### Population studies in neuroscience, blueberries, and anthocyanins

In a pooled analysis of 2 US cohort studies which examined almost 150,000 people, lower Parkinson disease risk was associated with the highest quintile of anthocyanin (RR: 0.76) and berry (RR: 0.77) intake (*P* = 0.02) (90). In a prospective analysis of 16,000 women in the Nurse's Health Study, greater intake of blueberries and strawberries was associated with slower rates of cognitive decline in older adults, with an estimated delay in decline of about 2.5 y (5).

The risk for Alzheimer disease and other dementias is associated with cardiovascular and metabolic health risk biomarkers, including obesity and insulin resistance in midlife (91–93). Inasmuch as anthocyanins are protective against CVD and T2DM risks, greater anthocyanin intake may be associated with reduced risk of Alzheimer-type dementia in late life.

### Clinical studies in neuroscience and blueberries

Cognitive performance in elderly adults improved after 12 wk of daily intake of blueberry (94) or Concord grape (95) juice. Better task switching and reduced interference in memory was found in healthy older adults after 90 d of blueberry supplementation (96). Blueberry powder intake led to modest benefits in memory performance and subjective improvements in everyday function among 39 older adults with cognitive complaints (97). These kinds of improvements reflected better executive ability (97). Interestingly, relatively modest benefits were found in cognitively unimpaired older adults (96, 97) compared with benefits measured in participants with mild cognitive impairment.

After 12 wk of blueberry consumption, greater brain activity was detected using magnetic resonance imaging in healthy older adults during a cognitive challenge. The detection was associated with enhanced perfusion in regions mediating cognitive function (98). Similarly, during a memory test, regional blood oxygen level-dependent activity detected by MRI (99) was enhanced in the subjects taking blueberry, but not in those taking placebo. All subjects in this study had mild cognitive impairment (99).

Cognitive benefits were detected in school-aged children in an acute study design where performance on a list-learning task was improved 2 h after consuming a single dose of blueberry powder but not placebo (100). Improvement in executive and long-term memory in children was associated with their intake of blueberry powder, with evidence of a dose-response (15 compared with 30 g powder) (101). In a crossover trial with children 7- to 10-y old, a single 30-g dose of blueberry powder produced enhanced executive performance on a timed and graded executive task (102).

Detecting cognitive benefits of blueberries in healthy children could be facilitated by tasks that involve a greater cognitive demand (102). Indeed, advancements in cognitive assessment tools will aid in examining specific populations. In particular, methods are needed to measure blueberry effects in cognitive domains involved in nonpathological aging, as opposed to domains affected by neuropathologies like Alzheimer disease. Statistical techniques such as covariate control and difference scores can help to identify the effects of phytochemicals like anthocyanins amid uncontrolled interindividual variation in factors such as cognitive capability, phase 2 metabolism, and intestinal microflora.

### Animal and mechanistic studies on blueberries and the brain

Blueberries improved cognitive and motor performance of aged rats, making them comparable to young animals (103, 104). Similar age-related improvements were observed in old mice (105). Blueberry-related improvements in long-term spatial memory of rodents is widely reported (29, 105–108). Cognitive benefits of blueberries in tasks that engaged working memory and learning are also documented (105, 108, 109).

Blueberry supplementation protected middle-aged mice from deficits in cognitive performance related to a high-fat diet (110). This is interesting in light of the rising incidence of obesity-related metabolic disorders (111) and the association between cardiometabolic markers in middle-aged humans and Alzheimer dementia risk later in life (91–93).

Blueberry supplementation protected vulnerable brain regions, reduced deficits in spatial memory, and mitigated markers of inflammation and oxidative stress in a rat model of accelerated aging due to high-energy particle exposure (112, 113). In a cell culture model of kainic acid–induced inflammation, treatment with blueberry polyphenolic fractions led to improved calcium buffering and reduced hippocampal neuron loss (114). Blueberry supplementation correlated with increases in hippocampal cAMP response element–binding protein phosphorylation and concentrations of brain-derived neurotrophic factor and improved performance in spatial working memory tasks of old animals (115).

Blueberry feeding is reported to upregulate neurogenesis, neuroplasticity, brain-derived neurotrophic factor, and insulin-like growth factor 1 in aged (106) and in young (107) rodents. Blueberry anthocyanidin glycosides and their phase 2 metabolites can cross the blood–brain barrier and are detectable in various brain tissues (24, 27–29, 116, 117).

## Blueberries and Anthocyanins in Vision and Eye Health

### Visual function, retinal stress, and anthocyanins

During vision, light reaching the eye is wavelength-filtered through the cornea, lens, and vitreous humor and focused onto the neural retina. Then retinal photoreceptors convert light energy into an electrical signal that is transmitted to the brain's visual centers via the axons of the retinal ganglion cells (RGCs).

The retina has the highest respiratory rate of any other mammalian tissue (118, 119) and is a significant source of oxidative stress. The outer segment of the retinal photo-receptor cell is rich in photopigments (opsin and 11-*cis* retinol) imbedded in membranes rich in polyunsaturated fatty acids which are constantly being renewed (120), thereby creating very favorable conditions for oxidative stress (121). Oxidative stress and cell proliferation are exacerbated by pathological responses to irradiation of the retina (122), neovascularization (123), and inflammation (124, 125). Markers of oxidative stress and inflammation increase with normal aging and can trigger a tissue-adaptive response (parainflammation) to restore homeostasis in the retina (126).

Although the retina is protected by an active blood–brain barrier at the retinal pigmentary epithelium (RPE), anthocyanins can be detected in ocular tissues. Anthocyanins were selectively distributed to ocular tissues after oral, intravenous, or intraperitoneal administration in rats and rabbits (26). In pigs fed diets containing 0%, 1%, 2%, or 4% (w/w) blueberries, anthocyanins were detected in the whole eye in a dose-dependent manner (127).

### Population studies on anthocyanins and vision

There are currently very few observational studies examining anthocyanin intake in relation to ocular disease risk. A higher total flavonoid intake was associated with a reduced risk of cataracts in a Finnish population of 10,054 subjects (128). In a prospective cohort study of >35,000 women aged ≥45 y, there was a significant association between blueberry intake and a reduced risk of incident total and visually significant age-related macular degeneration, but there was no association with incident cataract (H Sesso, Brigham and Women's Hospital, personal communication, 2019). Although macular degeneration is the leading cause of visual impairment during aging in the developed world, there are no studies that examine anthocyanin intake in relation to macular degeneration.

### Clinical studies on berry anthocyanins and vision

Compared to animal and in vitro research, there are relatively few clinical studies examining anthocyanin effects on human vision, particularly studies that adequately satisfy design criteria, including randomization, blinding, placebo control, and crossover, as previously described (129, 130). In normotensive glaucoma patients (*n *= 30), visual field defects were stabilized, ocular blood flow was improved, and plasma endothelin was normalized after 6 mo of daily intake of black currant anthocyanin (50 mg) (131). Similar benefits were observed in a trial in patients medicated for open-angle glaucoma, who received 25 mg anthocyanin daily for 2 y (132). Beneficial effects on intraocular pressure were also observed in a crossover study (*n *= 21) after only 4 wk of 50 mg daily intake (133).

Mirtogenol (bilberry and pine bark extract), corresponding to ~30 mg anthocyanin taken daily for 6 mo, provided additive benefit to ocular hypertensive patients (134), who were taking a widely used glaucoma treatment, prostaglandin F2a analog (Latanoprost) (135). The additive effect of Mirtogenol could have been due to normalization of capillary filtration, an antihypertensive effect related to vascular permeability. This effect was also suggested in a study of diabetic retinopathy patients using Tegens, a product similar to Mirtogenol (136). In a study of blueberries, the same protective effect was documented in an in vitro model of lipotoxicity-induced vascular endothelial dysfunction where greater NO bioavailability was linked to the blueberry effect (137).

An improvement in contrast sensitivity was associated with the intake of 510 mg bilberry anthocyanins daily in Tagen-F for 12 mo in human subjects with nonproliferative diabetic retinopathy (*n *= 88) (138). In a 1-mo crossover trial of 30 (139) and 60 normal subjects (140), anthocyanin intake was associated with an improved capacity for visual accommodation and a decrease in ocular fatigue of myopic subjects, possibly by improving contrast sensitivity.

Improvements were reported in dark adaptation threshold between highest dose and placebo, and visual accommodation shifts after a single dose ingestion of black currant concentrate at 12.5, 20, or 50 mg/dose (141). In two other recent crossover studies of normal-sighted adults (*n *= 60 and 72) there was no effect of blueberry juice intake on dark adaptation or dark-adapted visual acuity or contrast sensitivity, although a mild improvement in recovery time after retinal photobleaching was found (142). Interestingly, photobleaching recovery effects occurred with daily doses of either 7 or 346 mg blueberry anthocyanins and after both 3 and 12 wk of intake. In studies where low doses of anthocyanins were taken by healthy humans for a short term, there was no improvement in dark adaptation threshold, visual acuity, or contrast sensitivity (143–146), which conflicts with earlier research which reported such benefits (for review, see references 129 and 130).

### Blueberry and anthocyanin effects in animal models of vision

In studies using light-induced retinal photoreceptor degeneration, which is a widely used model of human retinal dystrophies (147), neuroprotection by blueberry species was convincingly documented with both long-term (5–35 d) (148, 149) and short-term (2–72 h) (149–153) prophylactic treatments with daily anthocyanin doses between 10 and 500 mg. Retinal inflammation, which is a hallmark of many ocular pathologies, was mitigated in mice fed bilberry extract (500 mg/kg BW) for 4 d after inflammation was induced by intraperitoneal injection of LPS (154). In the bilberry group, retinal electrophysiology was improved, rhodopsin was preserved, and there was less damage to photoreceptors compared to controls (154). In a similar model of retinal inflammation, mice fed for 5 d with 50–200 mg/d bilberry showed a dose-dependent decrease in neurotoxic NO and malondialdehyde, combined with an increase in neuroprotective antioxidant capacity due to glutathione, vitamin C, superoxide dismutase, and glutathione peroxidase (155).

Other ocular pathologies targeting primarily the RGC have also been investigated. The degeneration of RGC in vivo was mitigated with bilberry extract intake [100 mg/(kg ⋅ d)] in a mouse model of optic nerve injury. Bilberry extract (1%) mitigated RGC damage in vitro during oxidative conditions created with 3-(4-morpholinyl) sydnonimine hydrochloride (156). Bilberry also protected RGC of mice in vivo under oxidative conditions created by *N*-methyl-d-aspartic acid injected into the vitreous (20–100 µg/eye) (156).

Ocular development can be experimentally influenced by berry intake. When myopia was induced in young chicks by interposing a strong minus lens in front of the eye, the impact was less in chicks fed black currant extract (400 mg/kg BW) for 3 d prior to treatment (157). Retinal degeneration and cataract development were slowed with bilberry extract (20 mg/kg BW) in hypertensive OXYS rats that demonstrate senescence-accelerated expression of traits and a short lifespan (158). In neonatal rats where cataracts were induced by subcutaneous injection of sodium selenite, administration of a polyphenol-enriched fraction of bilberry at 40 mg/d was sufficient to prevent cataract formation (159). This effect was probably modulated through the regulation of nuclear factor erythroid 2–related factor 2 and hemoxygenase-1 in the lens (159).

Neonatal mice exposed to a high level of oxygen develop vascular complications similar to the retinopathy of prematurity in humans. Intraocular injection of bilberry extract (300 ng/eye) after neonatal oxygen exposure inhibited the formation of neovascular tufts by possible inhibition of vascular endothelial growth factor A and its downstream-regulated kinases (160).

### Blueberries, anthocyanins, and vision physiology examined in vitro

The in vitro antioxidative capacity of blueberries and their anthocyanins, used either prophylactically or as a treatment, has been demonstrated in vision-relevant models related to oxygen donation (161, 162), quenching of singlet oxygen (163), glutathione synthesis (149, 164), and glutamate insults (165) in both RPE and RGC primary culture cell lines.

The action of anthocyanins as molecular allosteric effectors has been investigated with the receptor protein rhodopsin (166) and with bestrophin, a protein involved in Best vitelliform retinal dystrophy (167). The allosteric actions of anthocyanins and flavonoids to inhibit cataractogenesis in vitro has been reported (168–171).

Bilberry anthocyanins improved viability and differentiation of cultured human corneal epithelial cells (172) and wild Chinese blueberry (*V*. *uliginosum* L.) produced similar benefits in the RPE cell line D407 (173). Blueberry treatment improved the viability and differentiation of human RPE cells during light-induced aging and after multiple replications in vitro (174).

Several studies document a potential role for flavonoids to improve retinal photoreceptor sensitivity in vitro by affecting the rate of rhodopsin regeneration (166, 175–177), or by modulating the inhibition of downstream G proteins involved in the phototransduction cascade (178, 179), or by downregulating retinoid-binding proteins (163). In an in vitro bovine ciliary muscle preparation, anthocyanins interacted with the endothelin-1 pathway to reduce muscle contractility, which relates to accommodative processes for distance vision in myopic eyes (180).

## Blueberries, Anthocyanins, and Food Processing

Fresh blueberries are delicate and often processed soon after harvest to preserve them. Individual quick freezing is a widely used means to preserve blueberries, to retain vitamin C, total phenolics, anthocyanins, and antioxidant capacity (181). The percentage loss of blueberry anthocyanins during −18°C storage was 12% after 10 mo of storage (181).

Dried blueberries can be stored at room temperature. Whereas conventional thermal dehydration can cause significant losses to anthocyanins (182), freeze-drying is an excellent means to remove water while preserving blueberry phytochemical quality (183). Freeze-dried blueberry powder loses anthocyanins in a temperature-dependent manner with a half-life of 139, 39, and 12 d when stored at 25, 42, and 60°C, respectively (184).

Radiant zone drying of blueberry extract did not affect anthocyanin or total phenolic content (185). Nonthermal technologies such as high pressure and pulsed electric fields used in conjunction with refrigerated storage helped to retain blueberry vitamin C, total phenolics, and anthocyanins immediately after processing (186).

Blueberries can be processed into shelf-stable products (e.g., canned fruit, juices, and preserves); however, processing can lead to changes in the phytochemical profile. During juice and purée processing, heat, oxygen, and enzymes can degrade blueberry phytochemicals, with greatest losses to vitamin C and anthocyanins. Blueberries are low in ascorbic acid and high in anthocyanins (187), and notably anthocyanins are readily degraded by ascorbic acid (188, 189).

Homogenization of whole blueberries leads to oxidative loss of anthocyanins, proanthocyanidins, and flavonols, due to polyphenol oxidase (190). Enzyme-catalyzed oxidative damage can be mitigated by blanching prior to milling and depectinization (191). Pasteurization to inactivate micro-organisms and enzymes typically results in minor (<10%) losses of blueberry polyphenolic compounds, although product flavor can be adversely affected (192). Polyphenolic compounds are lost when polyphenolic-rich skins and seeds in the press cake are physically removed (193–195).

Shelf-stable blueberry products like jam (196), juice (197), and extracts (198) can lose polyphenolic compounds when stored at ambient temperature whereas refrigeration mitigates losses. Blueberry processing can drastically change fruit composition; therefore, processing methods that optimize extraction and shelf stability of health-beneficial compounds are worthy objectives.

## Conclusions

Selected research documenting blueberries as a health-promoting food has been presented. Evidence supporting a role for blueberries and anthocyanins in human health is outlined according to human observational and clinical evidence, followed by mechanistic research using animal and in vitro models. Blueberry treatments generally produce larger effects in experimental models involving stress or disease risk.

The relative amount of evidence presented supporting cardiovascular, glucoregulation, neuroprotection, and vision benefits differs. For example, whereas there is abundant epidemiological evidence for the cardioprotective effects of blueberries and anthocyanins, epidemiological evidence for blueberry or anthocyanin benefits in human vision is lacking. And where there is substantial clinical evidence showing blueberry-related improvements in cognition and brain function, there is relatively little epidemiological evidence on anthocyanins in this area.

The anti-inflammatory, antioxidant, and vasoprotective effects of blueberry components together contribute to well-regulated glucose delivery to insulin-sensitive tissues and good metabolic function. Each of these aspects has implications in multiple areas of healthy aging. Notably, biomarkers of cardiometabolic dysfunction are associated with risk for vascular and Alzheimer-type dementia in late life (92, 93), which may be related to the mitigation of neuroinflammation.

Improvement in anti-inflammatory biomarkers associated with blueberry intake is supported by observational (8), clinical (48), animal (87), and in vitro (114) evidence. Anti-inflammatory and immune benefits of blueberries may involve mucin-associated and other colonic microbiota (67), which constitutes a new domain for berry health research.

Blueberry benefits have been observed in both short-term (see, for example, references 18, 78, and 100) and long-term human interventions (see, for example, references 76 and 94), which suggests multiple modes of action.

In blueberry health research, several important areas remain poorly understood. For example, the dose dependency of clinical effects is mostly unclear (18, 101, 142). The bioactivity of anthocyanin metabolites in vivo, both collectively and individually, is still mostly unknown, as is the importance to health of anthocyanins localized in tissues. Another important question is the relative bioactivity in the colon of phenolic breakdown products of blueberry anthocyanins compared with similar phenolic compounds from other plant foods in the diet. Notably, these gaps in knowledge do not detract from our ability to tap into blueberry health benefits by increasing public consumption.

This review of research findings will hopefully aid consumers, healthcare providers, and the food and health industry to understand the current state of knowledge on blueberries and health. It can be safely stated that daily moderate intake (50 mg anthocyanins, one-third cup of blueberries) can mitigate the risk of diseases and conditions of major socioeconomic importance in the Western world.

## Notes

The United States Highbush Blueberry Council (USHBC) offered support for this article by providing an honorarium to each author but had no role in the design and conduct of the review.

Author disclosures: AC, LRH, RZ-R, no conflicts of interest. AC acts as an advisor to the USHBC grant committee and has received research support from the USHBC. RK, WK, AJS, and FT have received research funding from the USHBC and have no conflict of interest.The USHBC is an agricultural federal research and promotion board established by the USDA. It was founded by and is funded by its members who are blueberry farmers, processors, and importers. The USHBC does not sell any product and operates with independent oversight from the USDA.

Abbreviations used: BW, body weight; CVD, cardiovascular disease; RGC, retinal ganglion cell; RPE, retinal pigmentary epithelium; T2DM, type 2 diabetes mellitus.
